# The exposure of the Great Barrier Reef to ocean acidification

**DOI:** 10.1038/ncomms10732

**Published:** 2016-02-23

**Authors:** Mathieu Mongin, Mark E. Baird, Bronte Tilbrook, Richard J. Matear, Andrew Lenton, Mike Herzfeld, Karen Wild-Allen, Jenny Skerratt, Nugzar Margvelashvili, Barbara J. Robson, Carlos M. Duarte, Malin S. M. Gustafsson, Peter J. Ralph, Andrew D. L. Steven

**Affiliations:** 1 CSIRO Oceans and Atmosphere, Hobart, Tasmania 7000, Australia; 2 Antarctic Climate and Ecosystems Co-operative Research Centre, Hobart, Tasmania 7000, Australia; 3 CSIRO Land and Water, Canberra, Australian Capital Territory 2601, Australia; 4 Red Sea Research Center, King Abdullah University of Science and Technology, Thuval 23955-6900, Kingdom of Saudi Arabia; 5 Plant Functional Biology and Climate Change Cluster (C3), Faculty of Science, University of Technology Sydney, Sydney, New South Wales 2007, Australia

## Abstract

The Great Barrier Reef (GBR) is founded on reef-building corals. Corals build their exoskeleton with aragonite, but ocean acidification is lowering the aragonite saturation state of seawater (Ω_a_). The downscaling of ocean acidification projections from global to GBR scales requires the set of regional drivers controlling Ω_a_ to be resolved. Here we use a regional coupled circulation–biogeochemical model and observations to estimate the Ω_a_ experienced by the 3,581 reefs of the GBR, and to apportion the contributions of the hydrological cycle, regional hydrodynamics and metabolism on Ω_a_ variability. We find more detail, and a greater range (1.43), than previously compiled coarse maps of Ω_a_ of the region (0.4), or in observations (1.0). Most of the variability in Ω_a_ is due to processes upstream of the reef in question. As a result, future decline in Ω_a_ is likely to be steeper on the GBR than currently projected by the IPCC assessment report.

## Abstract (web summary)

As the oceans become acidic, corals reefs are threatened, generating a need to understand the driving forces controlling the chemical state of the Great Barrier Reef. Here, the authors show a greater spatial variability than previously reported, created by the interaction of reef processes and ocean circulation.

## Graphical abstract

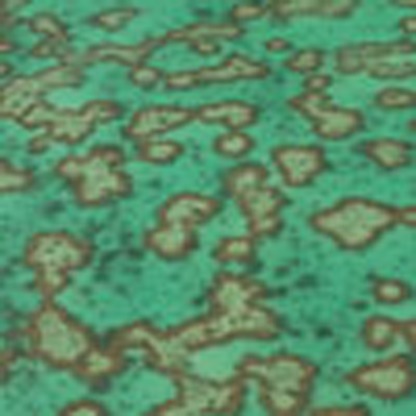

The Great Barrier Reef (GBR) ecosystem, described as one of the seven natural wonders of the world, is under increasing pressure from local and global anthropogenic stressors^1^. Coral calcification has continued to decline over the last few decades at rates similar to less well-managed reefs^2^, due to damage from cyclones, disease, invasive species (crown-of-thorns starfish), coral bleaching and possibly ocean acidification. In the long term, ocean acidification is expected to become an increasing threat to the GBR ecosystem^3^.

Ocean acidification is the decrease in pH and altered carbonate chemistry of ocean waters, including a decrease in aragonite saturation state of seawater (Ω_a_) due to the uptake of anthropogenic carbon dioxide that will impact the ability of many reef-building corals to precipitate CaCO_3_ (ref. 4). The Ω_a_ of tropical surface waters is predicted to decline by about 0.1 per decade over this century^5^. Informed decision making for the future management of the GBR in the face of global ocean acidification will necessitate that the current mean state, variability and drivers of Ω_a_ are known for individual reefs^6^,^7^.

The task to measure Ω_a_ at all individual reefs in the GBR (Fig. 1) is impossible. Earth System Models assessing current and future Ω_a_ (ref. 8) have spatial and temporal resolutions that are too coarse to resolve the variability in Ω_a_ in coastal and shelf environments, where vulnerable reefs, and a diversity of processes that contribute to regulating Ω_a_, exist^9^. Therefore, the current state of the carbon chemistry of the GBR system at the scale of individual reefs remains unknown.

The variability in Ω_a_ in coastal ecosystems is driven by complex interactions between forcing from the open-ocean carbon dynamics, the delivery of freshwater and carbon from coastal watersheds, and metabolic effects within the ecosystem^9^. Due to spatially and temporally variable processes affecting carbon chemistry, and the complex circulation of the GBR, the relative contribution of the drivers of Ω_a_ has not been well quantified for the region.

Observational studies on a small number of GBR reefs have shown that Ω_a_ is modified by processes such as calcification/dissolution and production/respiration on a reef^10^,^11^,^12^,^13^, and by the broader-scale impact of regional changes in carbon chemistry flowing onto each reef^8^,^14^,^15^. The relative contribution of the differing processes affecting Ω_a_ needs to be resolved to establish the vulnerability and likely impact on coral reef ecosystems to ocean acidification, and to incorporate ocean acidification into conservation and management strategies.

The novelty of the approach applied here allows us to estimate Ω_a_ and its drivers at over 3,000 reefs, a capability that is not possible using conventional approaches. A combination of total alkalinity (*A*_T_), dissolved inorganic carbon (*C*_T_), salinity, and temperature observations from 22 sites located inshore of the GBR (Supplementary Fig. 1), combined with a coupled catchment, hydrodynamic, sediment and biogeochemical model^16^,^17^,^18^ was used to estimate for the first time the Ω_a_, and the processes driving its variability, for the 22 coastal observations sites and 3,581 individual reefs (see Methods for model description). The observations are used to estimate Ω_a_ variability and to evaluate the approach and model performance.

The new maps quantifying the exposure of individual reefs to ocean acidification demonstrate a greater spatial variability than previous studies could resolve. The coupled model shows a clear spatial structure in Ω_a_, with large gradients across and along the GBR shelf, created by the interaction of reef processes and ocean circulation.

## Results

### Simulated ocean properties

In the biogeochemical model, the photosynthesis–respiration balance for planktonic and benthic (including coral) organisms is parameterized as a function of temperature, nutrients supply, light availability and predation control. Calcification–dissolution of calcium carbonate is a function of temperature, light, Ω_a_ and the type of benthic substrate. The model captures the heterogeneity of primary production on the GBR, with episodic riverine and upwelled shelf nutrient inputs driving local phytoplankton blooms along the shore and the continental shelf break^19^,^20^.

The modelled *A*_T_, *C*_T_, *S*, and Ω_a_, has temporal mean errors (root mean square errors, Supplementary Tables 1 and 2) at least a factor of 5 smaller than the observed spatial and temporal variability at the 22 observation sites, thereby providing confidence in the use of the model predictions (see Methods).

### Aragonite saturation-state variability

Across the 3,581 reefs, the model predicts an Ω_a_ that varies between 2.51 and 3.94 (Fig. 2a), (observations at 22 sites varied between 3.04 and 3.53, Table 1, Supplementary Fig. 1). Total alkalinity was generally below that of the Coral Sea mean, with values at the 3,581 reefs spreading from the mean open ocean (Fig. 2a). The southern GBR reefs showed a larger spread in *C*_T_ and *A*_T_ compared with the northern and central reefs regions (Fig. 2a).

A mean value of Ω_a_=3.61±0.19 (mean±s.e.) was estimated for the offshore Coral Sea source waters. The simulated Ω_a_ decreased towards the coast and was much lower in inshore reef waters (in areas <20 km from land, ΔΩ_reef–ocean_=−0.64, range=0.03 to −1.37, Fig. 2b), due primarily to higher *C*_T_. The outer reefs of the GBR (on the eastern side of the GBR lagoon) typically had Ω_a_ values above those of the mean Coral Sea (ΔΩ_reef–ocean_=0.12, range=−0.1 to 1.21), with the exception of the southern region, south of 20 **°**S (around the Swain Reefs).

### Drivers of aragonite saturation state variability

We considered three groups of processes driving change in the Ω_a_: freshwater fluxes from river flow, evaporation and precipitation and ocean circulation, referred to as the hydrological cycle component, ΔΩ_fresh_; net calcification, or calcification minus dissolution, ΔΩ_cd_; and the sum of photosynthesis minus respiration and net air–sea gas exchange, ΔΩ_pra_. The unique combination of impacts that each of these drivers have on temperature, *A*_T_, *C*_T_, and salinity (see Fig. 3 and Methods) enabled us to quantify the relative contribution of each driver to the change in Ω_a_ as waters flowed from the Coral Sea through the GBR shelf.

We found that freshwater fluxes (ΔΩ_fresh_) resulting from river flows, evaporation/precipitation, and oceanic circulation had limited influence on ΔΩ_reef–ocean_ (ΔΩ_fresh_<0.1 of ΔΩ_ocean–reef_) for most of the GBR reefs. The largest freshwater fluxes were observed in the southern outerreef region (near the Swain Reefs), south of 20**°** S (ΔΩ_fresh_=0.2), where ocean circulation alters the salinity of the region (Supplementary Fig. 2), with a corresponding change in Ω_a_. Net calcification and metabolic processes had a much greater impact on ΔΩ_reef–ocean_.

The change in Ω_a_ due to net calcification, ΔΩ_cd_, was generally negative, illustrating the importance of upstream net calcification in depleting carbonate ion concentration, thus reducing Ω_a_ at downstream reefs within the GBR (Fig. 4). The large negative ΔΩ_cd_ in the northern inner and southern GBR waters (−0.8<ΔΩ_cd_<−0.4 excluding the Capricorn Group) resulted from a strong net calcification signal in the outer reefs being transported into the northern and southern regions of the mid to inner shelf. In contrast, the outer reefs were flushed with relatively high Ω_a_ Coral Sea water.

The net balance of photosynthesis and respiration was a strong contributor to a downward shift in Ω_a_ (Fig. 4) as a consequence of respiration exceeding uptake of carbon due to photosynthesis, and a CO_2_ influx through air–sea gas exchange (Fig. 3). Metabolic effects were strongest (ΔΩ_pra_*=*−0.9) for the inner reefs, particularly for the central (14° S to 19° S) GBR (ΔΩ_pra_∼−0.4), compared with the southern and northern GBR reefs (ΔΩ_pra_ ∼ −0.2, Fig. 4). Positive ΔΩ_pra_ observed for most of the outer reefs suggested photosynthesis exceeds respiration in the water upstream of these reefs.

## Discussion

The results show Ω_a_ for an individual reef is strongly modulated by the circulation of waters and the regional balance of production/respiration, and dissolution/calcification processes. These processes cause the mean Ω_a_ value of reefs of the GBR to be 0.51 less than that of the open ocean. Further, the mean spatial variability in Ω_a_ within the GBR is large and is approximately equal to the temporal trend expected over the twenty first century under high carbon emission scenarios. As a result, we find that the projected Ω_a_ under all emissions scenarios will be lower on large parts of the GBR than estimated in the IPCC AR5 report^5^. This, combined with the projected changes in thermal stress^21^,^22^, suggests that even low future emissions scenarios may not be sufficient to avoid significant impacts of low Ω_a_ values of coral reef calcification with potential losses in coral cover, ecosystem biodiversity and resilience^23^.

The understanding of the regulation of Ω_a_ across the GBR provides new insights into how future environmental changes may impact ocean acidification on individual reefs. Changes in the hydrological cycle (for example, freshwater inputs) will have a limited impact, particularly as few reefs are located in regions where river plumes are frequent. The outermost reefs on the shelf edge are flushed with relatively high Ω_a_ waters from the Coral Sea, and currently net primary production counteracts and in some locations exceeds the decreases in Ω_a_ due to net calcification. As net calcification decreases on these reefs the influence of net primary production could result in Ω_a_ increases relative to Coral Sea values. For the inner to mid-shelf reefs, Ω_a_ decreases relative to upstream values. A source of *C*_T_ associated with net respiration or gas exchange causes a decline in Ω_a_, while net calcification in the North and South GBR causes further declines in Ω_a_. The model indicates that central reef waters may already be showing signs of dissolution in inter-reef regions exceeding the net calcification in the reefs and this region is likely to expand north and south with time. The balance of net calcification and primary production will be influenced by multiple factors that are difficult to predict.

Thus the future Ω_a_ regime of the GBR is dependent on the balance of calcification and primary production, which have different drivers and cannot be extrapolated directly from forecasts for the open ocean. Accurate forecasts of the exposure and vulnerability of GBR reefs to ocean acidification will require the future trajectories of regional carbon cycle and calcification/dissolution processes, both likely to also be impacted by ocean warming, and other anthropogenic pressures^14^.

The present methodology represents a step forward in our capacity to assess the contribution of various processes to Ω_a_ regulation in coastal ecosystems like the GBR. The lack of observations in carbon chemistry space still makes the validation of the model for the entire GBR difficult and this remains an important issue that needs resolution.

The quantitative understanding of the drivers of Ω_a_ provides insights that can be used to inform local management practices. The identification of regions with higher Ω_a_, combined with estimates of how the dominant drivers of Ω_a_ at these locations will likely be impacted by climate change, provides the best opportunity to identify zones on the GBR that will be least impacted by global acidification in this century. The approach developed here provides a pathway to advance beyond coarse ocean acidification models that focused on open ocean surface waters, to address the complex regional and local variability characteristic of the coastal ecosystems. In addition, the study offers a methodology and approach, based on a simple conceptual model (Fig. 3) that can be applied to other coastal ecosystems to elucidate key drivers of Ω_a_ variability.

## Methods

### Carbon chemistry calculations

Calcifying organisms utilize dissolved carbonate (CO_3_^2−^) and calcium (Ca^2+^) ions, to produce calcium carbonate (CaCO_3_) skeletal materials and shells. Aragonite is a metastable form of calcium carbonate that is precipitated biogenically by many reef forming corals and other species. The aragonite saturation state, Ω_a_, is commonly used to describe the ability of corals to calcify and is given by:


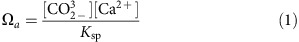


where *K*sp is the solubility product^24^. A fall in CO_3_^2−^ ion concentration is expected to cause a decrease in coral growth^25^.

The Ocean-Carbon Cycle Model Intercomparison Project (OCMIP) numerical methods are used to quantify air–sea carbon fluxes and the carbon dioxide system equilibria in seawater. The OCMIP procedures quantify the state of the CO_2_ system using two prognostic variables, the concentration of dissolved inorganic carbon, *C*_T_, and total alkalinity, *A*_T_. The value of these prognostic variables, along with salinity and temperature, are used to calculate the partial pressure of carbon dioxide, pCO_2_, in the surface waters using a set of governing chemical equations that are solved using a Newton–Raphson method^26^ with literature-derived constants^24^,^27^,^28^,^29^. One alteration from the typical implementation of the OCMIP algorithm is that we increased the search space for the iterative scheme from ±0.5 pH units (appropriate for global models) to ±2.5. This altered OCMIP scheme converges over the broader range of *C*_T_ and *A*_T_ values^30^, and is essential in our biogeochemical model that includes sources and sinks from tropical rivers, shallow coastal areas and offshore reefs. The air–sea flux of carbon dioxide is calculated based on 2-h wind speed data from the Australian Bureau of Meteorology operational forcing products, and the air–sea gradient in pCO_2_, using an empirical net flux relationship^31^.

Throughout the study, we assumed that in the region of interest, total alkalinity was approximated by carbonate alkalinity, hence neglecting the effect of biological nitrogen assimilation and release on *A*_T_. Wolf-Gladrow *et al*.^32^, showed that biological processes could be an important sink of *A*_T_ in some regions, but is a negligible term on the GBR shelf^33^.

### Separation of the drivers of change

The change in Ω_a_ over an individual reef from that of the open ocean, ΔΩ_reef–ocean_, can be broken down into the change due to three biogeochemical processes or drivers: freshwater fluxes from river flow, evaporation and precipitation, ΔΩ_fresh_; the sum of calcification and dissolution, ΔΩ_cd_, and the sum of photosynthesis, respiration and air–sea exchange of CO_2_, ΔΩ_pra_. This separation is possible due to the unique influence the drivers have on total alkalinity, *A*_T_, dissolved inorganic carbon, *C*_T_, and salinity, *S*, see Fig. 3 for details

Thus, we assume that change in Ω_a_ between the open ocean and the local reef, ΔΩ_reef–ocean_, is the sum of the three drivers:


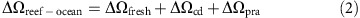


ΔΩ_reef–ocean_ is calculated using equilibrium carbon chemistry and the alkalinity and dissolved inorganic carbon in the open ocean (*A*_T(ocean)_, *C*_T(ocean)_, respectively) to determine the open-ocean aragonite saturation (Ω_ocean_), and that above the reef (*A*_T(reef)_, *C*_T(reef)_, respectively) to determine the reef aragonite saturation Ω_*reef*_ (at temperature 25 °C and salinity 35):


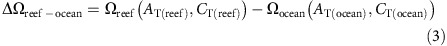


where *C*_T(ocean)_=1,966 μmol kg^−1^ and *A*_T(ocean)_=2,300 μmol kg^−1^ (Ω_a_=3.8) are climatological values from the Coral Sea^34^.

The model is forced with current, temperature and salinity at the ocean boundary by an ocean global circulation model^35^. *C*_T_ and *A*_T_ values at the ocean boundary are computed using existing salinity relationships^36^,^37^.


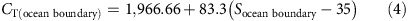



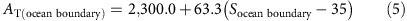


The salinity at the boundary varies temporally and spatially from 34.5 to 35.5; leading to a change in *A*_T_ from 2,278 to 2,331 μmol kg^−1^ and *C*_T_ from 1,880.3 to 2,008.4 μmol kg^−1^.

The variables *A*_T_, *C*_T_ and *S* are concentrations, independent of temperature. In the absence of biological processes and air–sea exchange, each is a conservative tracer. This property has been used to determine a climatology of *C*_T_ and *A*_T_ for the region:


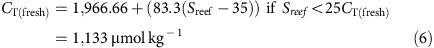



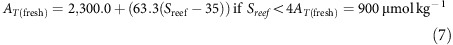


Using these values, the changes in Ω_a_ due to freshwater fluxes is given by:


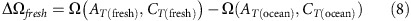


The salinity thresholds for *C*_T_ and *A*_T_ are different as we used the same value of 900 μmol kg^−1^ for freshwater *C*_T_ and *A*_T_.

Calcification and dissolution consume/produce twice as much *A*_T_ as *C*_T_. The remaining processes (photosynthesis, respiration and air–sea exchange) only change *C*_T_. Therefore, the change in the alkalinity from that of the reef, *A*_T(reef)_, from the value obtained considering only conservative fluxes, *A*_T(fresh)_, is due to calcification/dissolution only. Further, *C*_T_ must change by half this amount. Thus, the change in Ω_a_ from open ocean due to calcification and dissolution processes is:


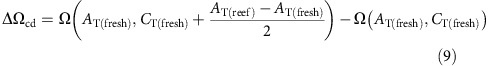


Finally, we can calculate the remaining driver:


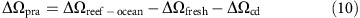


Where ΔΩ_pra_ is the change in Ω_a_ from open ocean due to photosynthesis minus respiration (by benthic and pelagic organisms), and includes net air–sea gas exchange driven by changes in the solubility of CO_2_ (a function of water temperature, salinity and pressure) and biological processes.

As *A*_T_, *C*_T_ and *S* are also available for the 22 observations sites, we use the same methodology to derive ΔΩ_inshore,obs-ocean_ and the corresponding drivers (presented in Supplementary Fig. 1).

To remove any bias from seasonal cycles, we averaged the model over a 2-year period (1 dry and 1 wet year) and validated the model using a 4-year hindcast. The spatial gradient ΔΩ_reef–ocean_ did not change seasonally (Supplementary Fig. 3), which indicates that the annual mean of this value is a good representation of the spatial structure.

### Skill of model predictions of carbon chemistry and validation

The skill of the model assessed at the 22 inshore observational sites provided a measure of the uncertainty of the Ω_a_ predictions at the 3,581 reef locations. The root mean square (r.m.s.) error of time series of *A*_T_, *C*_T_, *S* and temperature at each site was calculated (Supplementary Figs 4 and 5, Supplementary Tables 1 and 2). The mean (and range) of r.m.s. errors across the 22 sites were: *A*_T_: 39.90 mmol m^−3^(8.5, 91.5 mmol m^−3^); *C*_T_: 35.9 mmol m^−3^ (12.5, 63.97); *S*: 0.47 (0.15, 0.93); temperature: 0.87 °C (0.63, 1.24), resulting in an error in the calculated Ω_a_ of 0.23 (0.09, 0.54).

Due to their geographical locations, the observational sites are strongly influenced by freshwater plumes from rivers. The model is too coarse to resolve some of the small-scale water circulation features, such as internal waves, filament and small freshwater plumes. Freshwater footprints are difficult to accurately be represented in a 4-km resolution ocean model. For example, the real freshwater plumes could be thinner than the model grid cell, or could be offset in space and time, which make a comparison with observations at a point in space deceptive. Despite these problems, we are confident that the amount and frequency of freshwater inputs is correct (and has been validated in our model by many salinity observations), but their spatial footprint and timing could be offset.

Supplementary Fig. 6 shows, as an example, a point-by-point time series comparison at one of the inshore sites. Most of the variability at this location, for both the simulated and observed quantities, follows the seasonal cycle of temperature and the episodic flooding events (illustrated as freshwater input flow from the nearest river, as a green line on the salinity panel). The model reproduces baseline evolution of temperature, salinity, *C*_T_, *A*_T_ and Ω_a_. When a mismatch between observed and simulated entities occurs (like in April 2011), it usually follows a discrepancy in simulated salinity (most likely due to a different plume dynamics in the model compared to the reality, as mentioned above). Consequently, during these periods, simulated *C*_T_, *A*_T_ and Ω_a_ (representing a mean over a 4-km grid) are failing to get as low as the single-point observations.

This does not mean the model is incorrect, it just shows that we cannot always use a single point observation to validate the model. As long as the model is not used to predict the dynamic of a single point, the approach remains valid.

However, given the available observations, we show that our mechanistic model does simulate the Ω_a_ variability and its drivers at the 22 sites where we have sufficient carbon data to make the comparisons. Elsewhere, the assessment of other key features of the model, like the circulation, salinity and primary production show we do capture the spatial mean variability evident in the observations.

### eReefs hydrodynamic and biogeochemical model configuration

The model output is generated by the eReefs coupled hydrodynamic, sediment and biogeochemical modelling system^17^,^18^,^19^. The hydrodynamic model is nested within a global circulation model, to provide accurate forcing data along the boundary within the Coral Sea, and forced by atmospheric winds and radiation and 22 rivers flows. We used a 4-km resolution eReef grid^18^, and output from a hindcast from September 2010 to July 2014. The hydrodynamic model produces hourly fluxes between grid cells, which are used to determine the advection of the sediment and biogeochemical tracers.

The model is designed to simulate water chemistry around reefs and throughout the GBR reef matrix (4 km scale), but does not resolve processes inside the lagoon of the individual reefs (100 m scale). We use a complex biogeochemical model^38^ that simulates optical, nutrient, plankton, benthic organisms (seagrass macroalgae and coral), detritus, chemical and sediment dynamics across the whole GBR region, spanning estuarine systems to oligotrophic offshore reefs (Supplementary Fig. 7). Ocean acidification is quantified by the *A*_T_ and *C*_T_ (in turn determining pH and Ω_a_), that are altered by processes of calcification, carbonate dissolution, primary production, remineralization and gas exchange processes. The model also simulates the nutrient, sediment and freshwater inputs from the rivers system along the GBR.

The eReefs coral growth model considers corals and zooxanthellae as two separate entities, allowing corals to grow through both autotrophic and heterotrophic means^39^. Coral calcification rates are based on the biomass of corals and bottom light levels^40^. The calcification and reef dissolution rates were adapted from^10^,^11^, and are similar to those used in ref. 40. Sediment dissolution for grid cells on the continental shelf (depth<200 m) and between reefs was set to 0.0001, mmol C m^−2^ s^−1^. This constant rate of sediment dissolution was required to keep *A*_T_ from drifting below observations in coastal regions. For the purposes of predicting Ω_a_, the sediment dissolution rate was the least well-constrained parameter, and is likely to vary spatially and temporally. The current model does not consider calcite as another form of CaCO_3_, which are specific to the *Halimeda* beds, particularly in the northern GBR.

The GBRMPA Great Barrier Reef and Coral Sea Geomorphic Features data set identifies 3,860 individual reefs^41^. This database was used to set the initial distribution of corals in the model domain (3,581 reefs resolved). Due to the 4-km resolution of the model, in some instances >1 identified reef occurs within a single grid cell. Thus the 4-km model contains only 1,725 cells with reefs.

### Hydrodynamic-simulated GBR ocean circulation

**Figure 1:**
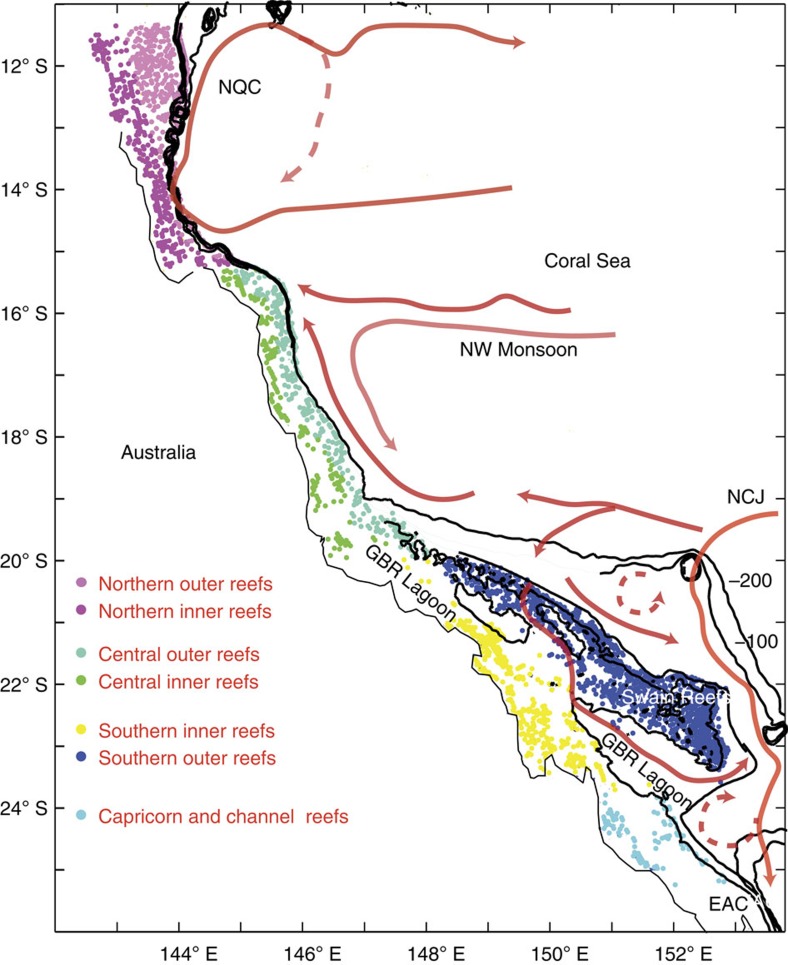
Great Barrier Reef living coral distribution. Black lines shows the 60, 100 and 200 m depth contours. Red lines show major surface currents. NQC: North Queensland Current; EAC: East Australian current; NCJ: North Caledonia Jet. Dashed orange lines show transient currents. The coloured dots show the individual reefs as identified by the Great Barrier Reef Marine Park Authority (GBRMPA) shelf features e-atlas separated into different geographical zones.

**Figure 2:**
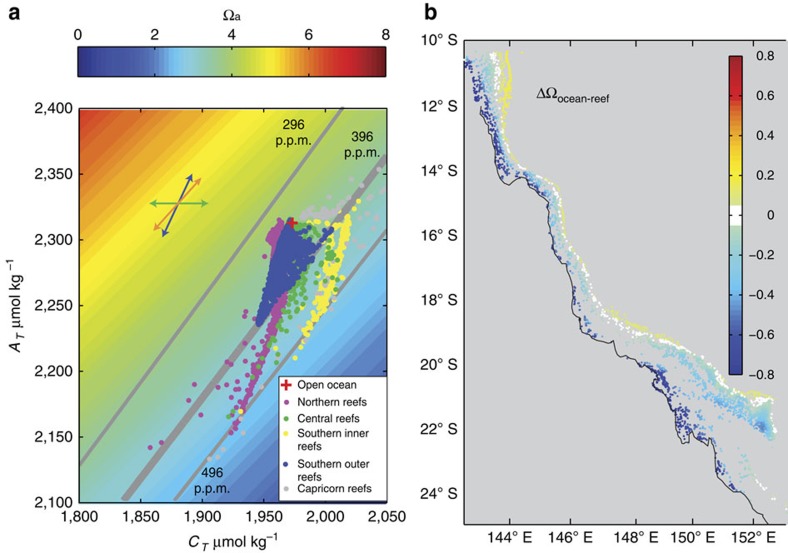
Aragonite saturation state for the 3581 coral reefs. (**a**) Simulated aragonite saturation state (as background shading) versus dissolved inorganic carbon, *C*_T_, and total alkalinity, *A*_T_, for the individual coral reefs (average from daily September 2010–September 2012 values). Grey lines show the surface pCO_2_ of 396 and 296 p.p.m. The Ω_a_ was calculated at a temperature of 25 °C and salinity of 35. If the Ω_a_ used as the background shading in **a** is calculated at 21 and then 27 °C, the range observed in the GBR surface waters, Ω_a_ at a constant *C*_T_ and *A*_T_ changes by <±0.07 from the shaded values. The process arrows are approximations that are further discussed in Fig. 3. (**b**) For the individual reefs the mean difference in aragonite saturation state between the open ocean value and the value simulated at the reef (ΔΩ_*ocean–reef =*_(Ω_ocean_−Ω_reef_).

**Figure 3:**
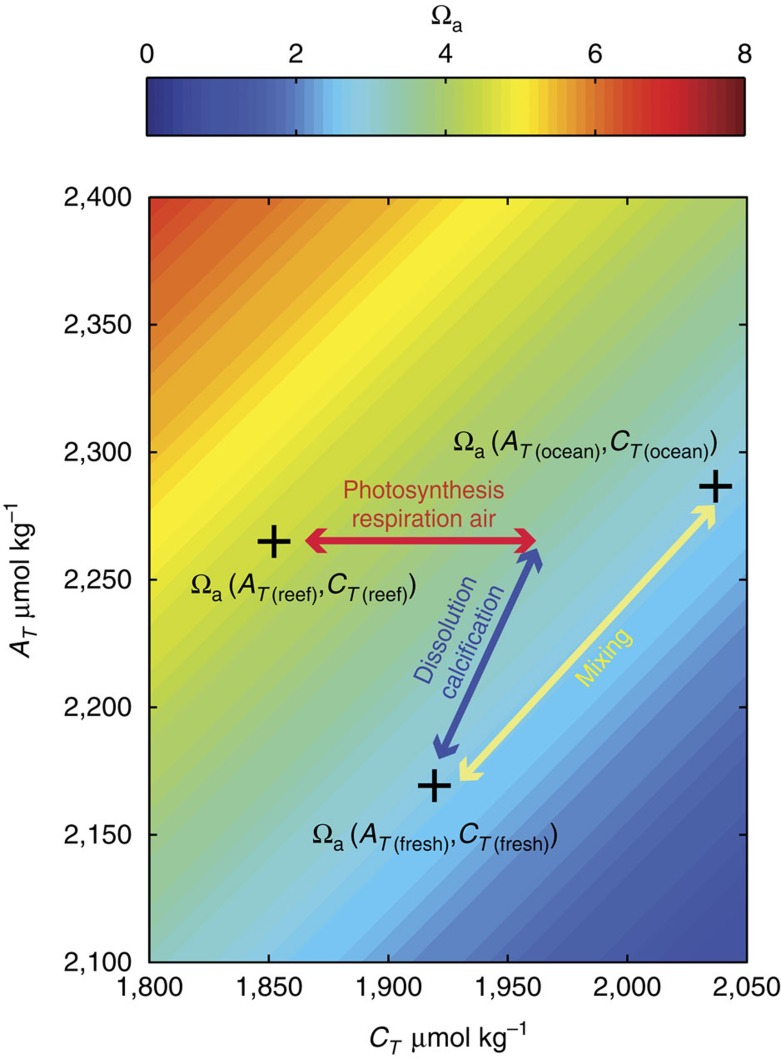
Driver of aragonite saturation-state change schematic. The change from the open ocean value is separated into three drivers: (1) hydrological cycle calculated considering dilution/mixing associated with a change in salinity (yellow arrow); (2) Net calcification is calculated as a shift in alkalinity and dissolved inorganic carbon at a ratio of 2:1 (blue arrow); (3) net carbon uptake due photosynthesis, respiration and air–sea flux (red arrow). Based on these values, the change in aragonite saturation from the open-ocean state is calculated. Background shows aragonite saturation-state versus dissolved inorganic carbon, *C*_T_, and total alkalinity, *A*_T_, at a temperature of 25 °C and salinity of 35.

**Figure 4:**
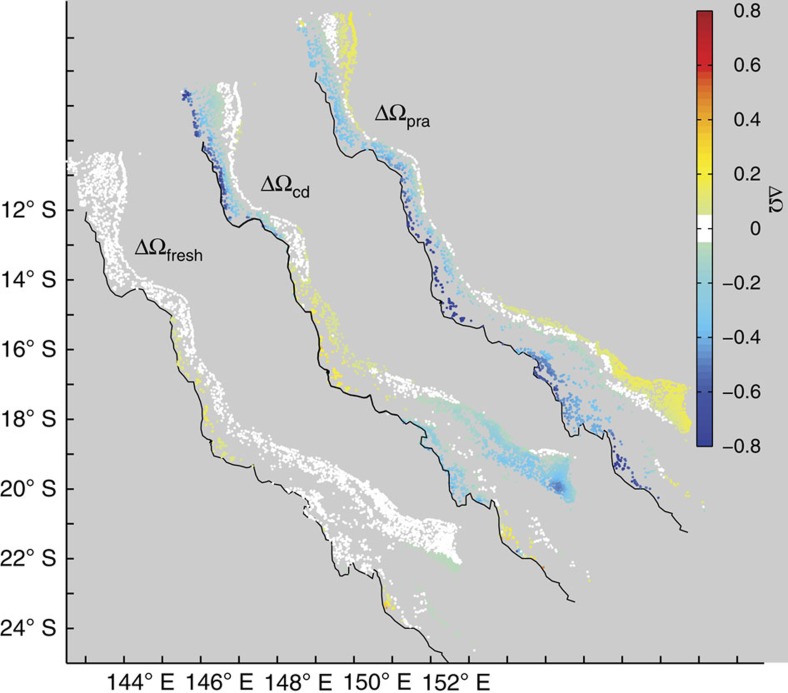
Drivers of change in aragonite saturation. The changes are separated into the affect due to hydrological cycle (ΔΩ_fresh_; left), net calcification (ΔΩ_cd_; centre), and due to the combination of photosynthesis, respiration and air–sea exchange (ΔΩ_pra_; right), average from daily September 2010–September 2012 values.

The general circulation of the GBR region is dominated by the westward flow of the South Equatorial Current flowing through the Coral Sea, as a number of water jets controlled by the complex plateau, seamount and ridge topography. On approaching the western boundary of the Coral Sea, these multiple jets are steered by the continental shelf to form the southward flowing East Australian Current and the northward flowing North Queensland Current (Fig. 1). Flow within the relatively shallow GBR lagoon is determined by the interaction of the above-mentioned basin-scale forcing, seasonal winds patterns, and the restricted flow through GBR outer reefs.

### Quantification of local processes using an age tracer

We defined local processes affecting the Ω_a_ of the waters overlying a reef to be those biogeochemical processes occurring on each individual reef with no contribution from upstream reefs. To quantify the relative importance of upstream versus local processes, we focused on the net calcification driver, assuming that the other carbon cycle processes and the hydrological cycle had an intrinsically regional impact. We computed the component of the change in Ω_a_ from the open-ocean values due to net calcification at the reef site (ΔΩ_cd,local_), using a simulated age tracer as a proxy for residence time above a reef^40^, and the average change in Ω_a_ due to net calcification on a well-studied GBR reef.

We carried out an age tracer experiment^42^,^43^ to diagnose the residence time of water on different reefs in the model. The age tracer concentration, *τ*, was initialized at 0 days within each reef. The age tracer was advected and diffused by the hydrodynamic model in a similar manner to salinity.

We allowed the age to decay off the reef so that water from one reef did not unduly impact on the age of adjacent reefs. This gave the local age. When on a reef grid cell, the age increased at the rate of :


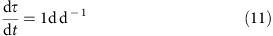


When the age tracer was not on a reef, age reduced at a rate proportional to its present age:


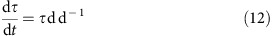


On the Heron Island reef, with an average depth of 2 m, a numerical simulation showed the local rate of change of Ω_a_ with age, is equal to 0.43 per day, similar to an observed value of 0.38 per day for the nearby One Tree Island reef^44^. For reefs with a depth of *h*, the local rate of change in Ω_a_ due to local net calcification in a model cell, using the Heron Island value, was given by:


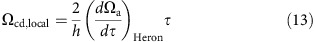


For the purposes of comparing local and upstream processes, we assumed all reefs had a mean depth of 2 m. Under this assumption, we overestimated the influence of local processes on reefs deeper than 2 m, and underestimated local processes for reefs shallower than 2 m. Local processes were shown at the scale of a reef (16 km^2^ in the model) to be small relative to the regional processes (Supplementary Fig. 8).

Results showed that the change in Ω_a_ due to local processes was almost negligible for the vast majority of the GBR, and only reached −0.05 in the inner northern GBR and Swain Reef regions where the largest reefs were present (Supplementary Fig. 1). While large variability in Ω_a_ is possible on sections of a reef, and will be significant for sub-reef scale community processes^45^,^15^, the mean Ω_a_ of an entire reef is generally forced by upstream processes.

### eReefs model description

The hydrodynamic model SHOC (Sparse Hydrodynamic Ocean Code^46^) was used in this study and is a general purpose model that is applicable on spatial scales ranging from estuaries to regional ocean domains. It is a three-dimensional finite-difference hydrodynamic model, based on primitive equations. Inputs required by the model include forcing due to wind, atmospheric pressure gradients, surface heat and water fluxes, and open-boundary conditions such as tides and low frequency ocean currents. The 4-km model is forced with OceanMAPS (http://www.bom.gov.au/bluelink/products/prod_oceanmaps.html) data on the open boundaries. The tide is introduced through 22 constituents derived from the global CSR tide model. The surface fluxes are obtained from ACCESS-R (http://www.bom.gov.au/nwp/doc/access/NWPData.shtml). Surface fluxes comprise momentum, heat and freshwater sources. Bathymetry has been be sourced from the Digital Elevation Model of the GBR produced at 100 m spatial resolution^41^. The model uses a curvilinear orthogonal grid in the horizontal and fixed ‘*z*' coordinates in the vertical.

The sediment transport model adds a multilayer sediment bed to the hydrodynamic model grid and simulates sinking, deposition and resuspension of multiple size-classes of suspended sediment^47^. The model solves advection-diffusion equations of the mass conservation of suspended and bottom sediments and is particularly suitable for representing fine sediment dynamics, including resuspension and transport of biogeochemical particles.

The model is forced with freshwater inputs at 21 rivers along the GBR and the Fly River. River flows input into the model are obtained from the Department of Environment and Resource Management gauging network (http://www.derm.qld.gov.au/water/monitoring/current_data). Relationships are used to account for nutrient and sediment inputs from rivers into the model (statistical relationships between river flow and nutrient concentrations)^18^,^19^,^48^.

The biogeochemical model is organized into three zones: pelagic; epibenthic; and sediment. The epibenthic zone overlaps with the lowest pelagic layer and the top sediment layer, sharing the same dissolved and suspended particulate material fields. The sediment is modelled in multiple layers with a thin layer of easily resuspendable material overlying thicker layers of more consolidated sediment. Dissolved and particulate biogeochemical tracers are advected and diffused throughout the model domain in an identical fashion to temperature and salinity. In addition, biogeochemical particulate substances sink and are resuspended in the same way as sediment particles. Biogeochemical processes are organized into pelagic processes of phytoplankton and zooplankton growth and mortality, detritus remineralization and fluxes of dissolved oxygen, nitrogen and phosphorus; epibenthic processes of growth and mortality of macroalgae, seagrass and corals; and sediment based processes of phytoplankton mortality, microphytobenthos growth, detrital remineralisation and fluxes of dissolved substances.

The biogeochemical model considers four groups of microalgae (small and large phytoplankton, *Trichodesmium* and microphytobenthos), three macrophytes types (seagrass species *Zostera* and *Halophila*, macroalgae) and coral communities. Photosynthetic growth is determined by concentrations of dissolved nutrients (nitrogen and phosphate) and photosynthetically active radiation. Autotrophs take up dissolved ammonium, nitrate, phosphate and inorganic carbon. Microalgae incorporate carbon (C), nitrogen (N) and phosphorus (P) at the Redfield ratio (106C:16N:1P), while macrophytes do so at the Atkinson ratio (550C:30N:1P). Microalgae contain two pigments (chlorophyll a and an accessory pigment), and have variable carbon:pigment ratios determined using a photoadaptation model.

Micro- and mesozooplankton graze on small and large phytoplankton, respectively, at rates determined by particle encounter rates and maximum ingestion rates. Half of the grazed material is released as dissolved and particulate carbon, nitrogen and phosphate, with the remainder forming detritus. Additional detritus accumulates by mortality. Detritus and dissolved organic substances are remineralized into inorganic carbon, nitrogen and phosphate with labile detritus transformed most rapidly (days), refractory detritus slower (months) and dissolved organic material transformed over the longest timescales (years). The production (by photosynthesis) and consumption (by respiration and remineralisation) of dissolved oxygen is also included in the model and depending on prevailing concentrations, facilitates or inhibits the oxidation of ammonia to nitrate and its subsequent denitrification to di-nitrogen gas, which is then lost from the system.

## Additional information

**How to cite this article:** Mongin, M. *et al*. The exposure of the Great Barrier Reef to ocean acidification. *Nat. Commun.* 7:10732 doi: 10.1038/ncomms10732 (2016).

## Supplementary Material

Supplementary Information Supplementary Figures 1-8 and Supplementary Tables 1-2.

## Figures and Tables

**Table 1:** Mean change in aragonite saturation state.

	Model mean (range)	Observations mean (range)	Model - Observations
Ω_reef_	3.14 (2.46–3.47)	3.21 (3.04–3.53)	−0.07
ΔΩ_reef–ocean_	−0.59 (−1.26, −0.28)	−0.52 (−0.71, −0.20)	−0.07
ΔΩ_fresh_	0.01 (−0.05, 0.12)	0.05 (−0.03, 0.14)	−0.04
ΔΩ_cd_	−0.20 (−0.57, 0.07)	−0.07 (−0.14, 0.17)	−0.13
ΔΩ_pra_	−0.40 (−0.82, −0.20)	−0.48 (−0.93, -0.11)	0.08

Average ΔΩ_reef–ocean_ and the drivers of this change, at the 22 observation sites (15 locations—some locations have multiple depths), as determined from model output and observations^5^ using *A*_T_, *C*_T_, *S*, and temperature. The number of observation points in time at each site ranges from 6 to 41. We compared model outputs of a 4-year hindcast (2010–2014), taken the day and hour of the observations, with the observation. Discrepancies between model and observations are not unexpected as the model represents the mean properties in a 4 by 4-km region, compared to a single observation point, in a highly dynamic coastal environment.
