# Supplementary material for: The exposure of the Great Barrier Reef to ocean acidification
Source: Nat Commun. 2016 Feb 23;7:10732. doi: 10.1038/ncomms10732 (PMC4766391; doi:10.1038/ncomms10732)
Supplement: Supplementary Information — Supplementary Figures 1-8 and Supplementary Tables 1-2. [file ncomms10732-s1.pdf]

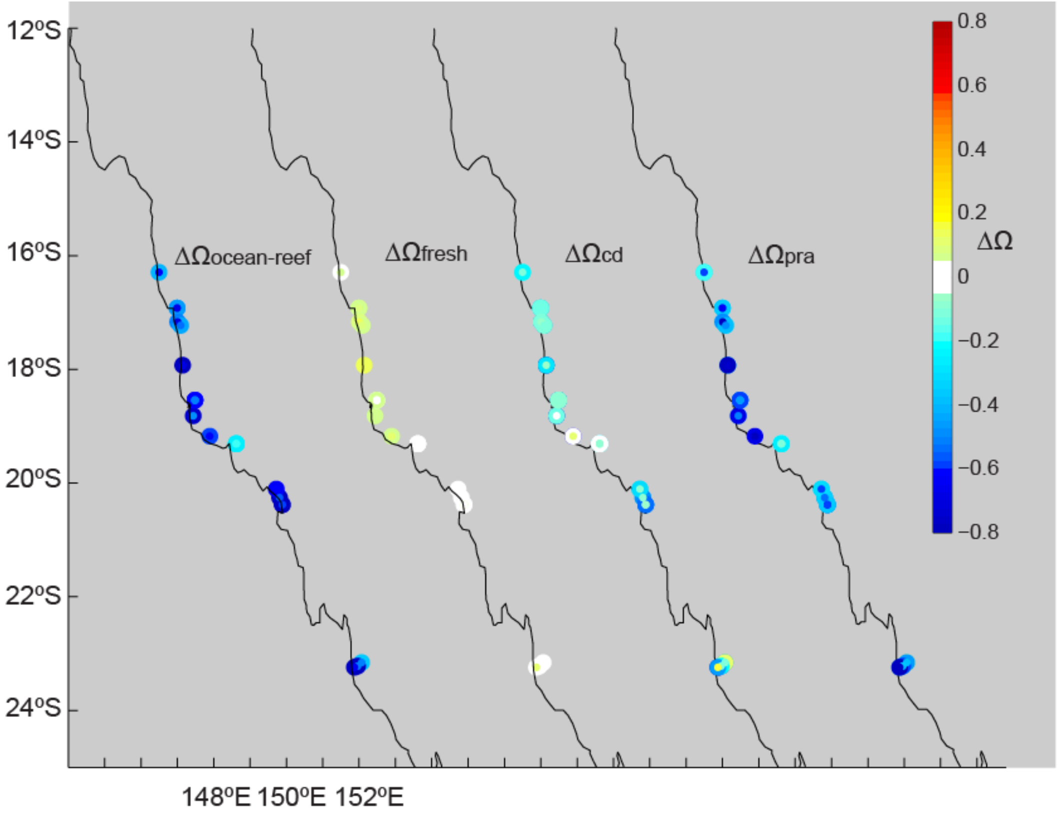

Supplementary Figure 1. Observed Aragonite saturation variability and its drivers. Mean shift in aragonite saturation state from open ocean values, $\Delta\Omega_{ocean\text{-}reef}$ (left), due to freshwater fluxes, $\Delta\Omega_{fresh}$ (2nd from left), net calcification, $\Delta\Omega_{cd}$ (2nd from right), and due to the combination of photosynthesis, respiration and air-sea exchange, $\Delta\Omega_{pra}$ (right) at sampling sites along the GBR. Values computed using the model outputs at the time of the observations were taken are shown as large circles. Values computed using observations are shown as small circles within the large circles symbol. These values were computed using observations at the regular sampling sites within the GBR and the equivalent model outputs. The model was able to predict the fraction of each of the drivers of $\Delta\Omega_{reef\text{-}ocean}$ at the 22 samples sites with an accuracy of approximately 0.2 (Table 1), giving confidence in the skill of quantify individual drivers at the 3,581 reefs.

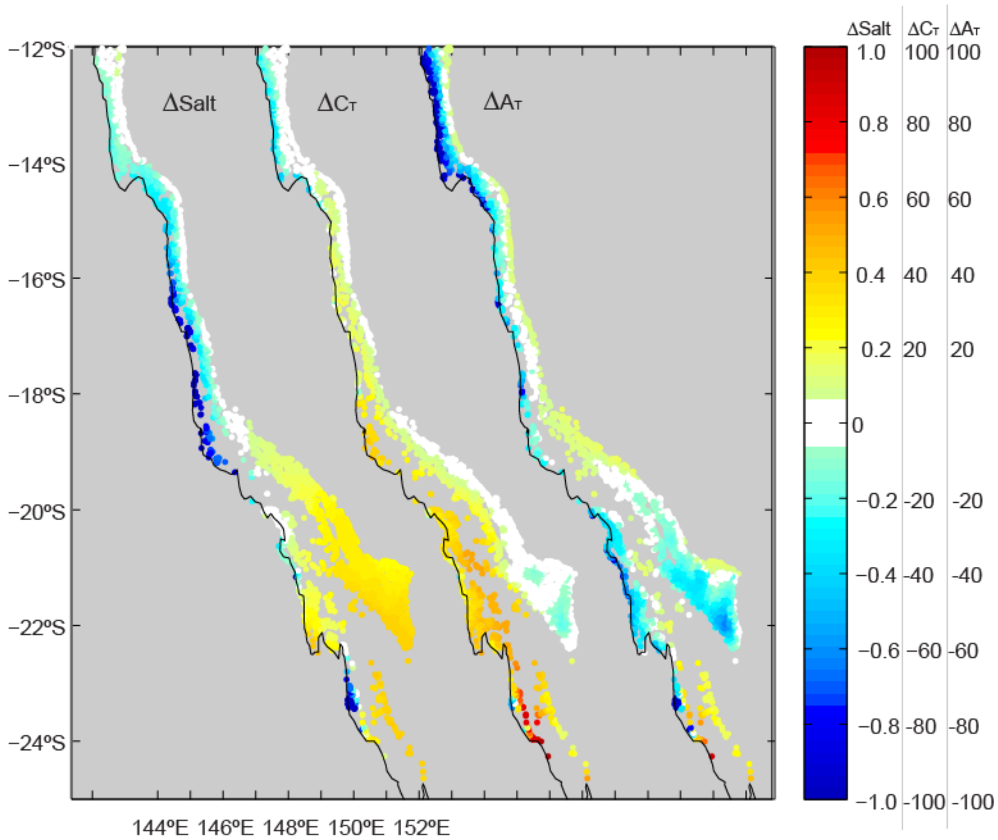

Supplementary Figure 2. Mean ocean properties. Mean (Sept 2010–Jul 2014) shift in surface salinity, total dissolved inorganic carbon, C_T, (μmol kg^{-1}) and total alkalinity, A_T (μmol kg^{-1}), from the mean Coral Sea values on 3,581 GBR reefs. The colour scaling of the three panels is given as three labels for each colour level.

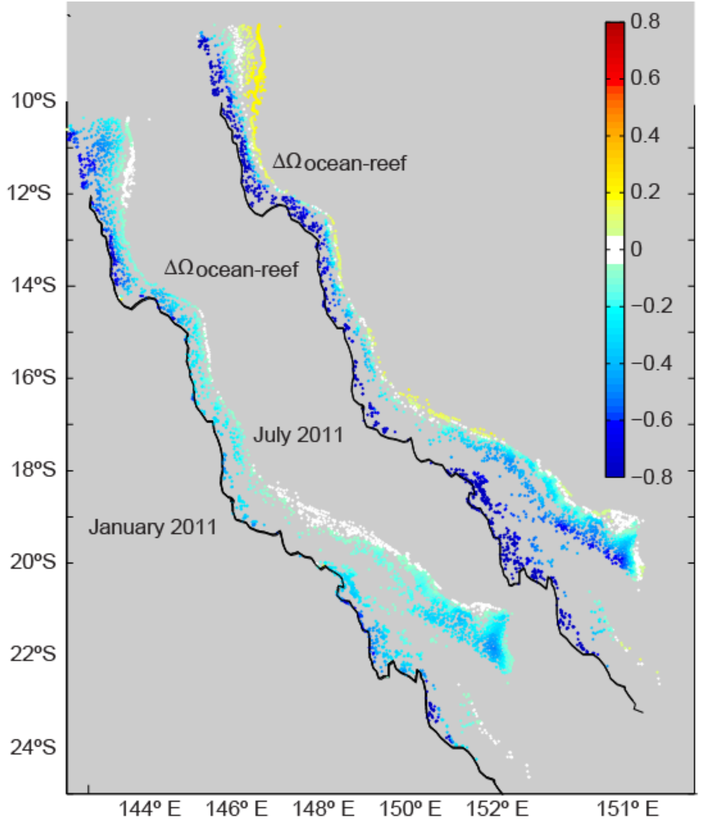

Supplementary Figure 3. Seasonal variability in Aragonite saturation state. $\Delta\Omega_{ocean\text{-}reef}$ for the 3,581 individual coral reefs of the Great Barrier Reef in January 2011 (left) and July 2011 (right).

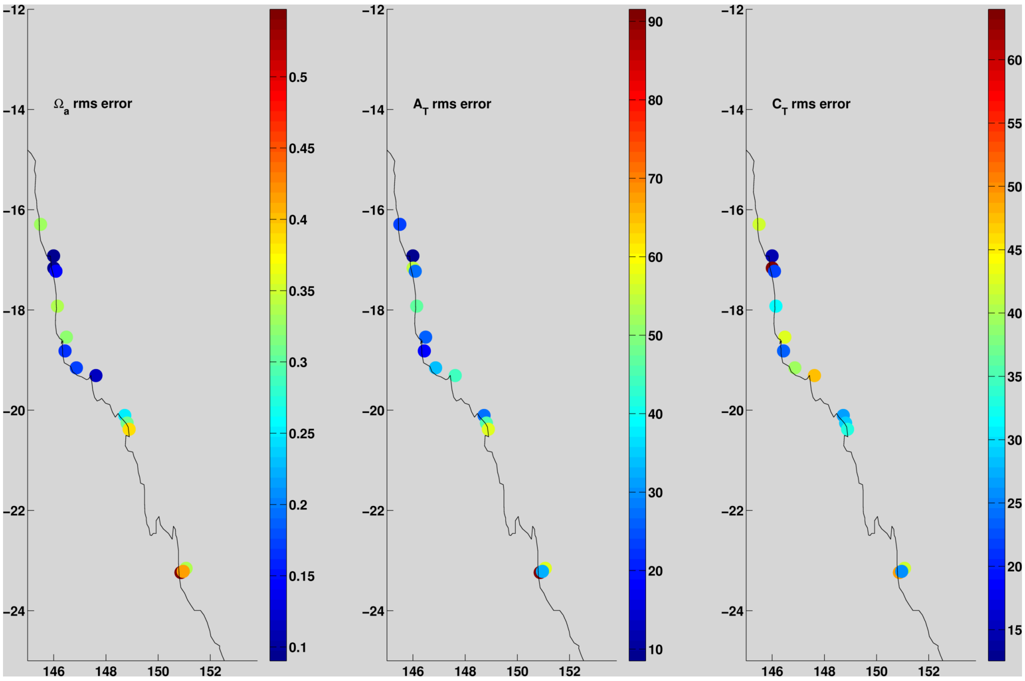

Supplementary Figure 4. Model root mean square error. Maps of root mean square (rms) error of time-series of simulated Ω_a, A_T (µmol kg^{-1}) and C_T (µmol kg^{-1}). Statistical values were computed using observations at the regular sampling sites within the GBR and the equivalent model outputs from the Sept. 2010–Jul 2014 period (these value are also reported in Supplementary Table 1).

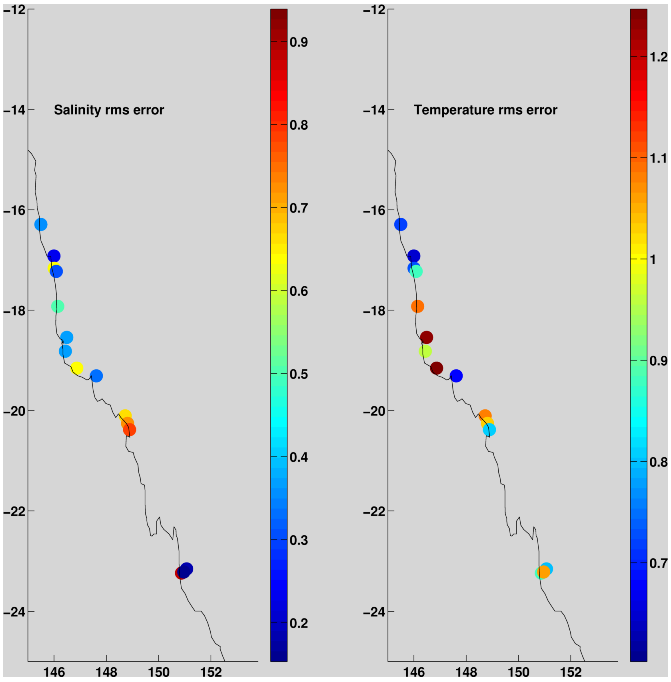

Supplementary Figure 5. Model root mean square error. Maps of root mean square (rms) error of time-series of simulated salinity and temperature (°C). Statistic values were computed using observations at the regular sampling sites within the GBR and the equivalent model outputs for the Sept. 2010–Jul 2014 period.

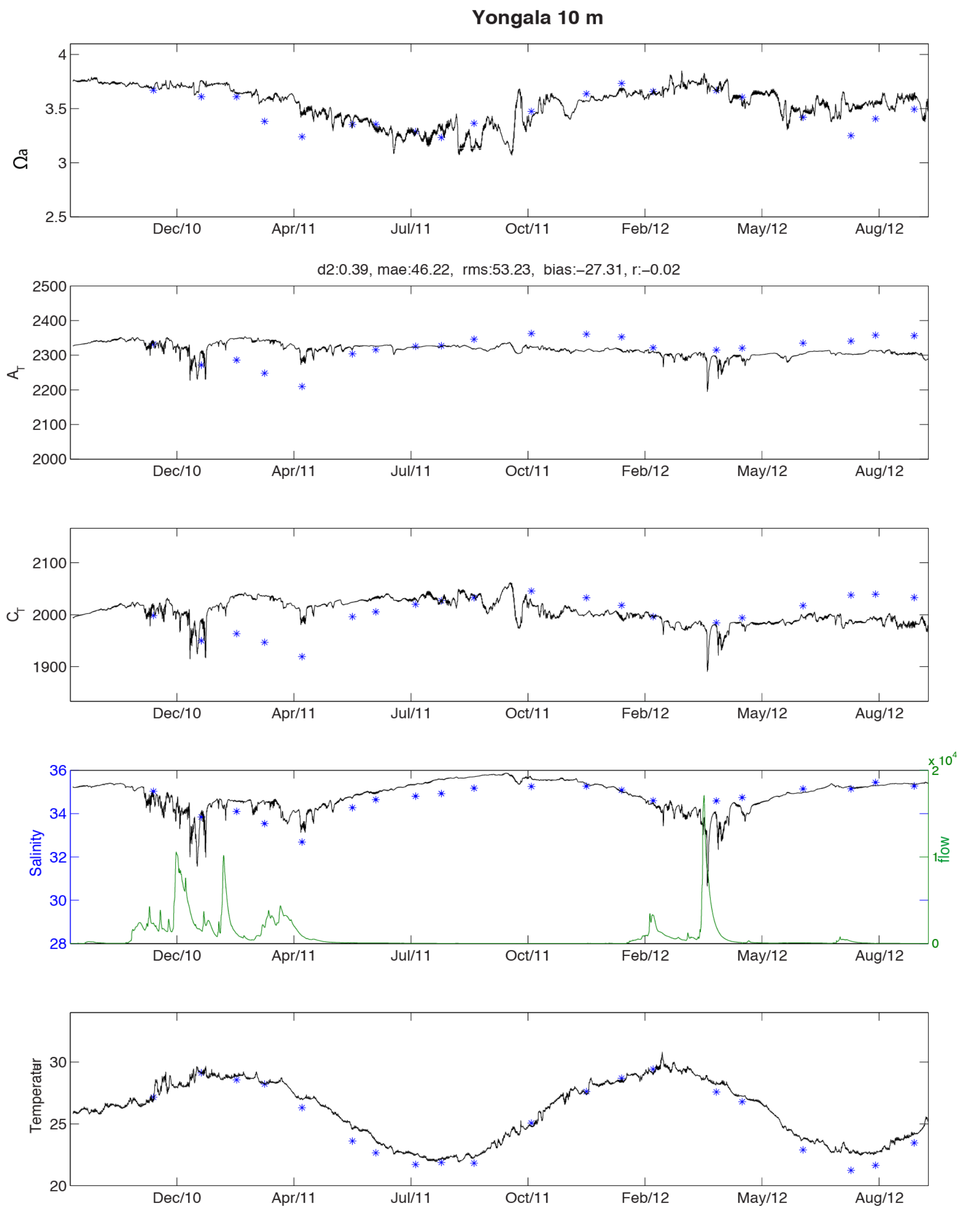

Supplementary Figure 6. Temporal evolution of ocean properties. Time series of Ω_a, A_T, T_C, salinity (with river flow in m^3 s^{-1} on the right axis) and temperature at the IMOS (Integrated Monitoring Observation System) National Reference Station of Yongala (NRSYON, Latitude: -19.305, Longitude: 147.622, depth 10m) Queensland, Australia. Observations are shown as blue stars representing monthly bottle sample, model is shown as daily outputs.

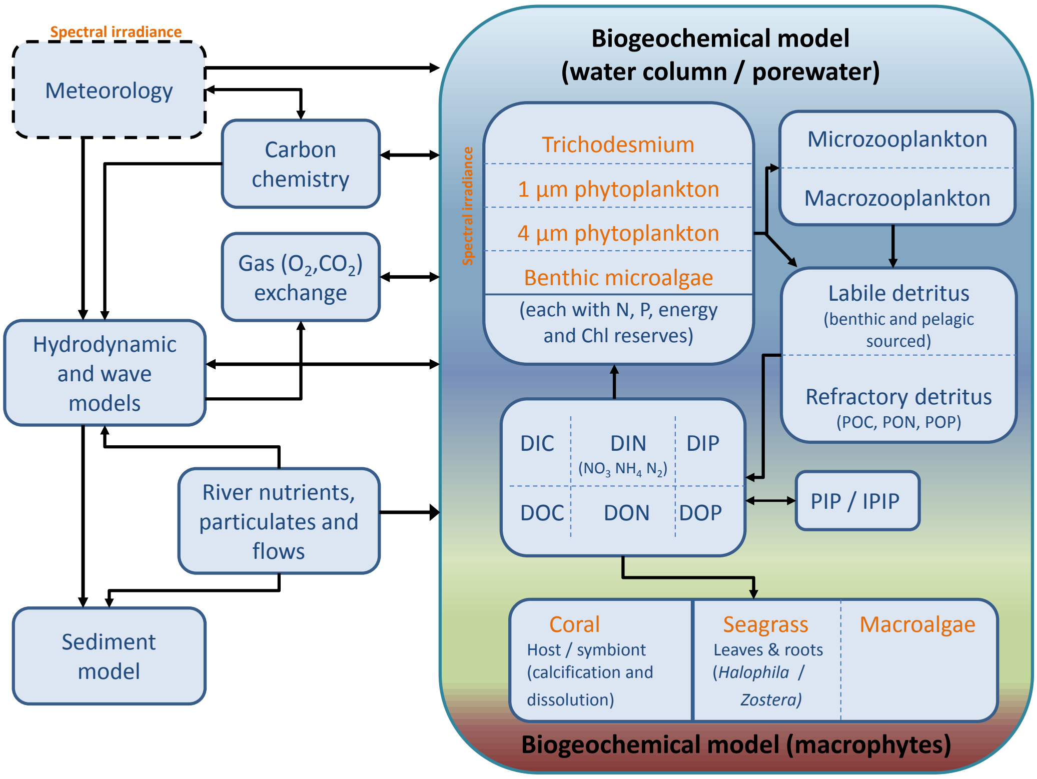

Supplementary Figure 7. Biogeochemical model schematic.

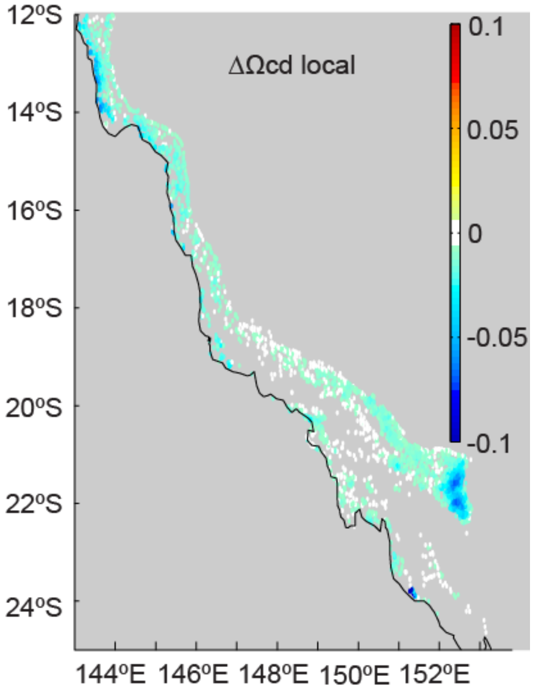

Supplementary Figure 8. Local processes. Mean (Sept. 2010–Jul 2014) shift in aragonite saturation state from open ocean values on the 3,581 GBR reefs due to local calcification and dissolution processes $\Delta\Omega_{cd,local}$.

Observations sites	$A_{T\ model}$	$A_{T\ obs}$	$A_{T\ model}$ - $A_{T\ obs}$	$C_{T\ model}$	$C_{T\ obs}$	$C_{T\ model}$ – $C_{T\ obs}$	Ω_{model}	Ω_{data}	Ω_{model} - Ω_{data}
**1**	2330.22	2292.83	37.38	2037.83	2008.08	29.75	3.33	3.23	0.10
**2**	2227.82	2270.42	-42.60	1967.76	1990.25	-22.49	2.94	3.17	-0.23
**3**	2254.67	2270.17	-15.49	1975.79	1992.67	-16.87	3.15	3.14	0.00
**4**	2180.33	2228.25	-47.92	1933.66	1956.79	-23.14	2.78	3.06	-0.27
**5**	2251.20	2260.08	-8.88	1953.57	1975.00	-21.43	3.33	3.21	0.12
**6**	2251.43	2241.25	10.18	1954.15	1968.25	-14.10	3.33	3.08	0.25
**7**	2279.64	2271.81	7.83	2002.25	2000.62	1.64	3.15	3.08	0.07
**8**	2242.26	2227.58	14.67	1951.18	1957.33	-6.15	3.26	3.04	0.22
**9**	2282.03	2299.14	-17.11	2035.52	2017.86	17.66	2.84	3.20	-0.36
**10**	2249.77	2271.38	-21.60	1987.53	1986.38	1.15	2.98	3.22	-0.24
**11**	2204.66	2284.25	-79.59	1992.05	2015.79	-23.74	2.46	3.06	-0.60
**12**	2251.64	2272.92	-21.28	1975.26	1984.83	-9.58	3.12	3.25	-0.13
**13**	2252.97	2272.92	-19.95	1976.66	1984.83	-8.17	3.12	3.25	-0.13
**14**	2252.61	2270.58	-17.97	1975.33	1984.92	-9.59	3.13	3.22	-0.10
**15**	2215.36	2271.40	-56.04	1964.83	2001.50	-36.67	2.84	3.07	-0.23
**16**	2220.04	2275.00	-54.96	1967.63	1998.38	-30.76	2.86	3.14	-0.27
**17**	2256.30	2245.83	10.46	1958.01	1963.25	-5.24	3.34	3.18	0.17
**18**	2246.18	2250.46	-4.28	1949.14	1976.88	-27.73	3.33	3.09	0.23
**19**	2298.35	2269.41	28.94	1990.59	1958.93	31.67	3.47	3.48	-0.01
**20**	2301.00	2276.33	24.66	1992.92	1963.41	29.51	3.47	3.51	-0.03
**21**	2304.06	2281.95	22.11	1996.03	1966.90	29.13	3.47	3.53	-0.06
**22**	2305.28	2282.32	22.95	1997.34	1967.04	30.30	3.47	3.53	-0.06

Supplementary Table 1. Temporal mean differences between simulated, and observed. A_T, C_T . (μmol kg^{-1}), and Ω_a at the 22 observation sites.

	$A_{T\ model}$	$A_{T\ obs}$	$A_{T\ model}$ - $A_{T\ obs}$	$C_{T\ model}$	$C_{T\ obs}$	$C_{T\ model}$ – $C_{T\ obs}$	Ω_{model}	Ω_{data}	Ω_{model} - Ω_{data}
**mean (22 obs sites)**	2257.17	2267.56	-10.38	1978.86	1982.72	-3.86	3.14	3.22	-0.07
**std (22 obs sites)**	35.54	18.17	30.79	25.51	18.47	21.78	0.27	0.15	0.21
**mean (3,591 reef sites)**	2286.80			1971.50			3.47		
**std (3,591 reef sites)**	31.43			20.81			0.298		

Supplementary Table 2. Mean differences between simulated and observed. A_T, C_T, and Ω_a at the 22 observation sites and simulated mean of A_T, C_T, and Ω_a at 3800 reef sites.
